# Using *Haloarcula marismortui* Bacteriorhodopsin as a Fusion Tag for Enhancing and Visible Expression of Integral Membrane Proteins in *Escherichia coli*

**DOI:** 10.1371/journal.pone.0056363

**Published:** 2013-02-15

**Authors:** Min-Feng Hsu, Tsung-Fu Yu, Chia-Cheng Chou, Hsu-Yuan Fu, Chii-Shen Yang, Andrew H. J. Wang

**Affiliations:** 1 Institute of Biological Chemistry, Academia Sinica, Taipei, Taiwan; 2 Core Facilities for Protein Structural Analysis, Academia Sinica, Taipei, Taiwan; 3 Institute of Bioinformatics and Structural Biology, National Tsing Hua University, Hsinchu, Taiwan; 4 Department of Biochemical Science and Technology, National Taiwan University, Taipei, Taiwan; 5 Institute of Biochemical Sciences, National Taiwan University, Taipei, Taiwan; University of Oulu, Finland

## Abstract

Membrane proteins are key targets for pharmacological intervention because of their vital functions. Structural and functional studies of membrane proteins have been severely hampered because of the difficulties in producing sufficient quantities of properly folded and biologically active proteins. Here we generate a high-level expression system of integral membrane proteins in *Escherichia coli* by using a mutated bacteriorhodopsin (BR) from *Haloarcula marismortui* (*Hm*BRI/D94N) as a fusion partner. A purification strategy was designed by incorporating a His-tag on the target membrane protein for affinity purification and an appropriate protease cleavage site to generate the final products. The fusion system can be used to detect the intended target membrane proteins during overexpression and purification either with the naked eye or by directly monitoring their characteristic optical absorption. In this study, we applied this approach to produce two functional integral membrane proteins, undecaprenyl pyrophosphate phosphatase and carnitine/butyrobetaine antiporter with significant yield enhancement. This technology could facilitate the development of a high-throughput strategy to screen for conditions that improve the yield of correctly folded target membrane proteins. Other robust BRs can also be incorporated in this system.

## Introduction

Integral membrane proteins constitute an important class of proteins with which are often involved in diverse biological functions, including G-protein coupled receptors (GPCRs), channels, transporters and enzymes. Approximately 20–35% of the open reading frames (ORFs) in the human genome are predicted to encode membrane proteins [1]. Membrane proteins account for more than 50% of current drug targets [2], [3]. Structural information about these pharmaceutically useful membrane proteins will assist in the design of better drug molecules. However, despite the need for identifying membrane protein structures, there are significantly fewer structures available for membrane proteins than for soluble proteins [4]. Some of the major hurdles associated with membrane protein purification include the production of insufficient yields and the inability to obtain diffraction quality crystals.

During the protein expression process, the problem of protein aggregation often arises in the form of inclusion bodies. To produce soluble cytosolic proteins, affinity/fusion tags are efficient tools for promoting the isolation of soluble proteins and facilitating the purification process. These tags allow protein purification without prior knowledge of the target proteins’ biochemical properties. Some common fusion tags include thioredoxin (Trx), maltose-binding protein (MBP), glutathione S-transferase (GST), intein, calmodulin-binding protein (CBP), NusA, and cellulose-associated protein (CAP) [5], [6]. A visible soluble protein expression system, the Cherry^TM^Express, utilizes a heme that binds to a portion of cytochrome and can be measured at 413 nm as a red fusion tag [7]. MBP and GST have been used to produce some integral membrane proteins but in low yield [8]. Mistic, a *Bacillus subtilis* protein, is a membrane-integrating protein that can assist in the folding of integral membrane proteins autonomously into the membrane and it has been used as a fusion tag for heterologous membrane protein expression in *Escherichia coli* (*E. coli*) with a culture yield of 1 mg/L [9]. Neophytou et al. [10] reported that *E. coli* glycerol-conducting channel protein (GlpF), a protein with eight-transmembrane helices, could be used as a fusion partner for eukaryotic integral membrane proteins expressed in *E. coli*, with a yield of 7.5 mg/L culture [11]. Green fluorescent protein (GFP) fused at the C terminus of the target proteins has been used as a detection probe for the development of high-throughput membrane protein screening [12]. For structure determination, a fusion partner (e.g., T4 lysozyme, flavodoxin, xylanase, rubredoxin or cytochrome b_562_RIL) was inserted into the third intracellular loop of GPCR, facilitating the crystallization of GPCRs [13].

Bacteriorhodopsins from photoreceptive archaebacteria, often found in hypersaline environments (3–5 M) such as salt lakes, salt ponds, and marine salterns are among the best-studied integral membrane proteins. Thus far, the complete genome of four such species has been sequenced: *Halobacterium salinarum* [14], *Haloarcula marismortui* [15], *Haloquadratum walsbyi* [16], and the haloalkaliphile *Natronomonas pharaonis* [17]. From genomic analysis, it can be seen that their metabolism is considerably different from each other based on their acidic protein machineries, respiratory chains and rhodopsins [18]. In *H. salinarum*, the sole bacteriorhodopsin protein (*Hs*BR) occupies nearly 75% of the cell surface area, forming a hexagonally symmetric purple membrane composed of three identical protomers. The *Hs*BR structure has been determined at high resolution by x-ray diffraction, which revealed seven transmembrane α-helices and one bound retinal molecule covalently linked to Lys216 forming the Schiff base [19]–[21]. After absorbing a photon, the retinal is isomerized from the all-*trans* to the 13-*cis* configuration. To investigate the photocycle of *Hs*BR, site-directed mutagenesis of aspartate residues at positions 85, 96, and 212 demonstrated that these mutants reduced the proton pumping activity [22]. Asp96 serves as the internal proton donor in the reprotonation of the Schiff base, and the D96N mutation increases the M state decay rate [23]. The relatively well-established experimental protocols associated with *Hs*BR allow us to consider other BRs for structural analysis as well as their possible use as protein carriers. Recently, Yang and his colleagues have cloned and expressed six putative photoreceptor proteins from *H. marismortui* in *E. coli* C43(DE3) [24]. *Hm*BRI/D94N, which is the corresponding residue for D96N in *Hs*BR, exhibits the same photochemistry properties (Fu et al. unpublished data, 2012). D96 in *Hs*BR is a critical residue for proton transportation, and D96N shows a weaker pH dependent proton pump activity. Surprisingly, the expression level of *Hm*BRI/D94N (40–70 mg/L culture) is higher than the wild-type *Hm*BRI (3–10 mg/L culture).

In this work, we designed systems in which the target membrane protein is fused with *Hm*BRI/D94N incorporating a histidine purification tag (His-tag) plus a Tobacco Etch Virus protease (TEVp) cleavage site at the N terminus of target protein. The undecaprenyl pyrophosphate phosphatase (UppP) and carnitine transporter (CaiT) of *E. coli* were chosen as the target membrane proteins to demonstrate the utility of our design. We have successfully purified milligram quantities (per liter of *E. coli* culture) of active UppP and CaiT integral membrane proteins, as judged by SDS-PAGE, matrix-assisted laser desorption/ionization mass spectrometry (MALDI-MS), functional assays and crystallization studies. Therefore the structurally robust and biochemically stable *Hm*BRI/D94N is a suitable fusion membrane protein fusion tag for the large-scale preparation of integral membrane proteins. With further modification of our system, new platforms can be tailored for high-throughput analysis, e.g., by incorporating parallel cloning vectors that use additional fusion tags.

## Results

### Selection of Bacteriorhodopsin

**Figure 1:**
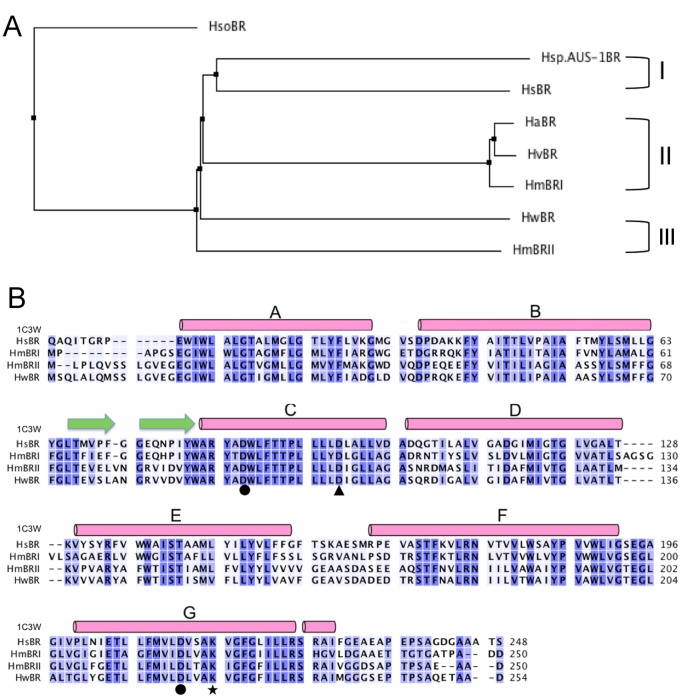
Phylogenic tree and multiple sequence alignment of bacteriorhodopsins. (A) Phylogenic tree of haloarchaeal bacteriorhodopsins. (B) *Hs*BR (PDB code: 1C3W), *Hm*BRI, BRII and *Hw*BR are aligned using ClustalW [41]. The seven-transmembrane helices are labeled A to G. The proton acceptor residues (Asp) are labeled with circles. The position of *Hm*BRI/D94N is labeled with a triangle, and the residue Lys bound to retinal by Schiff base interaction is labeled with a star.

**Figure 2:**
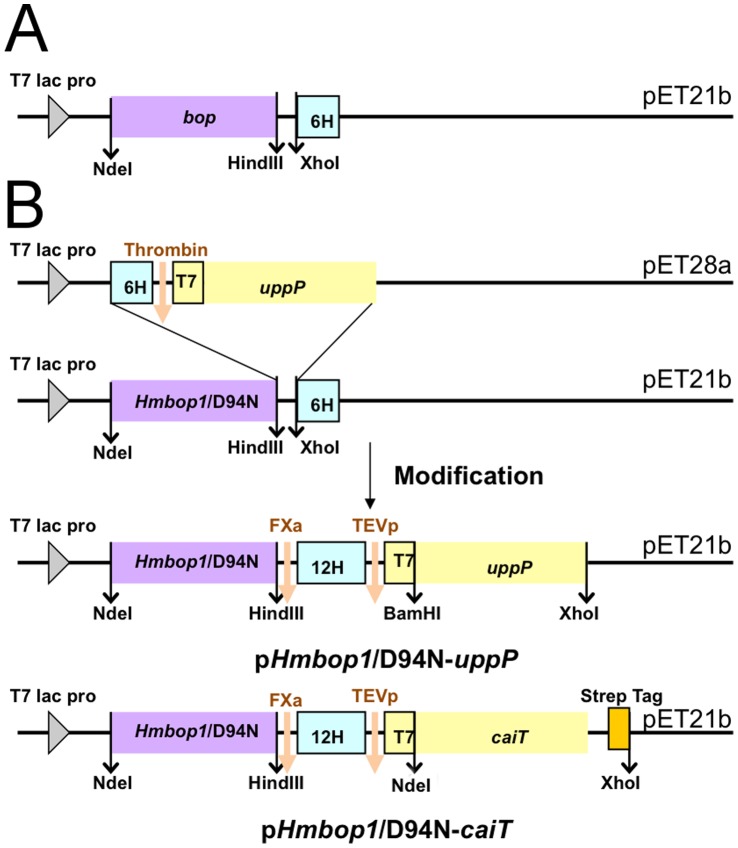
Schematic illustration of the cloning strategy for the p*Hmbop1*/D94N-*uppP* systems. (A) All the bop genes were constructed into pET21b with C terminal (His)_6_ tag. (B) The target gene, *E. coli uppP* gene was constructed into pET28a with 6×His tag, thrombin cleavage site and T7 tag at 5′ end. *Hmbop1*/D94N (*bop1* is the gene of BRI) was constructed in pET21b with a 3′ end-6×His tag. The 6×His-thrombin cleavage site-T7 tag-*uppP* fragment constructed in pET28a was then subcloned into the 3′ end of *Hmbop1*/D94N. After several modifications, p*Hmbop1*/D94N-*uppP* was generated for fusion protein expression. For construction of p*Hmbop1*/D94N-*caiT*, a Strep tag was inserted at the 3′ end of the *caiT* gene.

Haloarchaeal bacteriorhodopsins (BRs) are well-known purple integral membrane proteins. Furthermore, BR is stable above 80°C and under pH conditions ranging from 1.0 to 12.0 in an aqueous environment [25], [26]. The retinal chromophore is bound tightly to the Lys in the pocket. There are several BRs divided into three groups (Figure 1A), even though they have similar sequences (Figure 1B). We chose to study the protein expression of *Hs*BR, *Hm*BRI and *Hw*BR in *E. coli* and characterize their biophysical properties. In this study, BRs were cloned into pET21b with a 3′ end-6×His tag and were overexpressed in *E. coli* C43(DE3) (Figure 2A). *Hs*BR is the best-studied BR with low expression levels in the membrane fraction with *E. coli* as the host. In addition, the structures of *Hs*BR and its mutants purified from the native source have been solved [19], [27].

**Figure 3:**
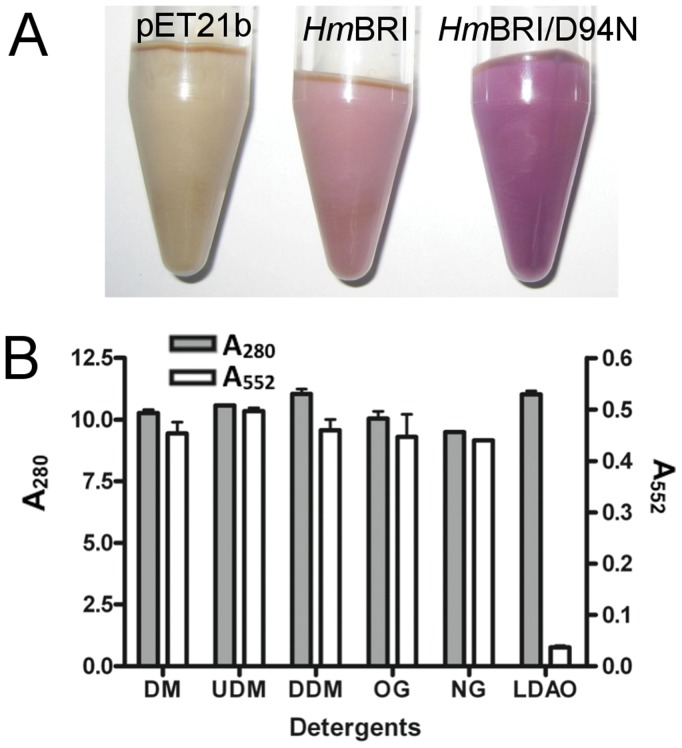
Expressed *E. coli* cell pellets and detergent screening of *Hm*BRI/D94N. (A) Expressed ell pellets induced by IPTG and retinal of *E. coli* C43(DE3) transformed with pET21b, *Hm*BRI and D94N. (B) Various common detergents including DM, UDM, DDM, OG, NG and LDAO were tested. Gray bars represent the total protein (A_280_) of detergent extracted membrane fractions and the open bars represent the specific absorption of *Hm*BRI/D94N (A_552_) of detergent extracted membrane fractions.

The sequence alignment of *Hs*BR, *Hm*BRI, *Hm*BRII and *Hw*BR showed that they share about 50% identities. Lys216 bound to retinal and Asp85, 96, and 212 of *Hs*BR that are important for proton transfer are conserved (Figure 1B). Fu et al [24] successfully expressed six photorhodopsins from *H. marismortui* with high expression levels using pET21b as the expression vector and *E. coli* C43(DE3) as the host, and analyzed their functions. *Hm*BRI/D94N can be used to study the slow down of the photocycle rate. When *Hm*BRI/D94N is expressed with *all-trans* retinal, we found that it had a high expression level (up to 40–70 mg/L culture), resulting in a dark purple cell pellet (Figure 3A). The high-level expression, stability and visible purple pellet of *Hm*BRI/D94N suggested that we can use *Hm*BRI/D94N as a tool for targeting integral membrane proteins to the membrane.

### Construction of the BR Fusion System with Different Target Membrane Proteins

The strategy for the construction of the BR fusion system is shown in Figure 2B. The target gene, *E. coli uppP*, was constructed in pET28a with a 6×His tag, a thrombin cleavage site and a T7 tag at the 5′ end. Because *Hm*BRI/D94N can be expressed well in pET21b with C-terminal 6×His tag, we designed primers with specific restriction enzyme sites and subcloned the thrombin cleavage site-T7 tag-*uppP* fragment, constructed from pET28a, into the 3′ end of *Hmbop1*/D94N. First, we introduced a Factor Xa cleavage site (IEGR↓) between *Hmbop1*/D94N and the His tag. Elongation of a 6×His into a 12×His tag can enhance the binding affinity of the fusion protein on a Ni-NTA column. TEVp is a commonly used protease with high specificity for cleaving the affinity tag from the target proteins [28]. Here, we substituted the thrombin cleavage site for a TEVp cleavage site (ENLYFQ↓G) to prepare the native target protein. If high-throughput screening of the target protein is required, the T7 tag (11 amino acids) generated from pET28a serves as a linker for the binding space of TEVp. The length of the linker can be adjusted depending on the characteristics of each protein.

To increase the purity of CaiT, a Strep purification tag was inserted at the C terminus of CaiT for further purification.

### Expression and Purification of the Proteins

To avoid the toxicity associated with the over-expression of heterologous membrane proteins in *E. coli*, we used mutant *E. coli* strains, C41(DE3) and C43(DE3), which are routinely used as host cells for successful expression BR membrane protein [29]. In our strategy, we established the same expression and purification steps for all of the constructs in order to consistently compare the expression level of each sample; however, the expression conditions for each construct could be tailored specifically designed to obtain the maximal expression yield.

All of the proteins in this research were expressed according to the same general protocol. When the cell density of each culture reached an OD_600_ of 1.0, protein expression was induced with 0.5 mM isopropyl-β-D-thiogalactoside (IPTG) for 5 h at 37°C in the presence of 5–10 µM *all-trans* retinal. *Hm*BRI and mutant D94N were cloned into pET21b with a C-terminal 6×His tag and expressed in *E. coli* C43(DE3). The expressed *E. coli* cell pellets can be easily detected visually (Figure 3A). pET21b, *Hm*BRI and D94N expressed in *E. coli* C43(DE3) adding *all-trans* retinal resulted in white, purple and dark purple cell pellets.

To purify *Hm*BRI/D94N, we treated the cell lysate in 50°C water bath for 30 mins, based on the heat stable property of BR after cell disruption [30]. The pellet containing the membrane fraction was extracted with n-decyl-β-D-maltoside (2%, DM) followed by Ni-NTA column purification, and *Hm*BRI/D94N was eluted with 500 mM imidazole. We obtained approximately 70 mg/L culture of purified *Hm*BRI/D94N for crystallization.

Detergents are critical components for extracting membrane proteins. To search for the proper detergents in purification of *Hm*BRI/D94N and to improve the wide-range usage of the fusion tag system, several common detergents are used to extract the *Hm*BRI/D94N protein. DM, UDM (n-undecyl-β-D-Maltoside), DDM (n-dodecyl-β-D-maltoside), OG (n-octyl-β-D-glucoside), NG (n-nonyl-β-D-glucoside) and LDAO (lauryldimethylamine-N-oxide) have different critical micelle concentrations (CMCs) and chemical and physical characteristics and are used to test the solubility of active *Hm*BRI/D94N. The yields of detergent solubilization can be estimated by A_280_ (total proteins) and A_552_ (functional *Hm*BRI/D94N) in the detergent-solubilized membranes (Figure 3B). The results show that these six detergents can solubilize an equal amount of proteins in the membrane fraction. The active *Hm*BRI/D94N protein can be solubilized by most detergents except LDAO, and the protein dissolved in DM, DDM and OG has proton pump activity [24]. The use of LDAO may cause the *Hm*BRI/D94N to fold like opsin, without bound retinal, which might be quenched by LDAO. DM, UDM, DDM, OG and NG can be used in suitable buffer conditions for extracting the *Hm*BRI/D94N fusion proteins for subsequent purification of the active target proteins.

### Characterization of BRs

**Figure 4:**
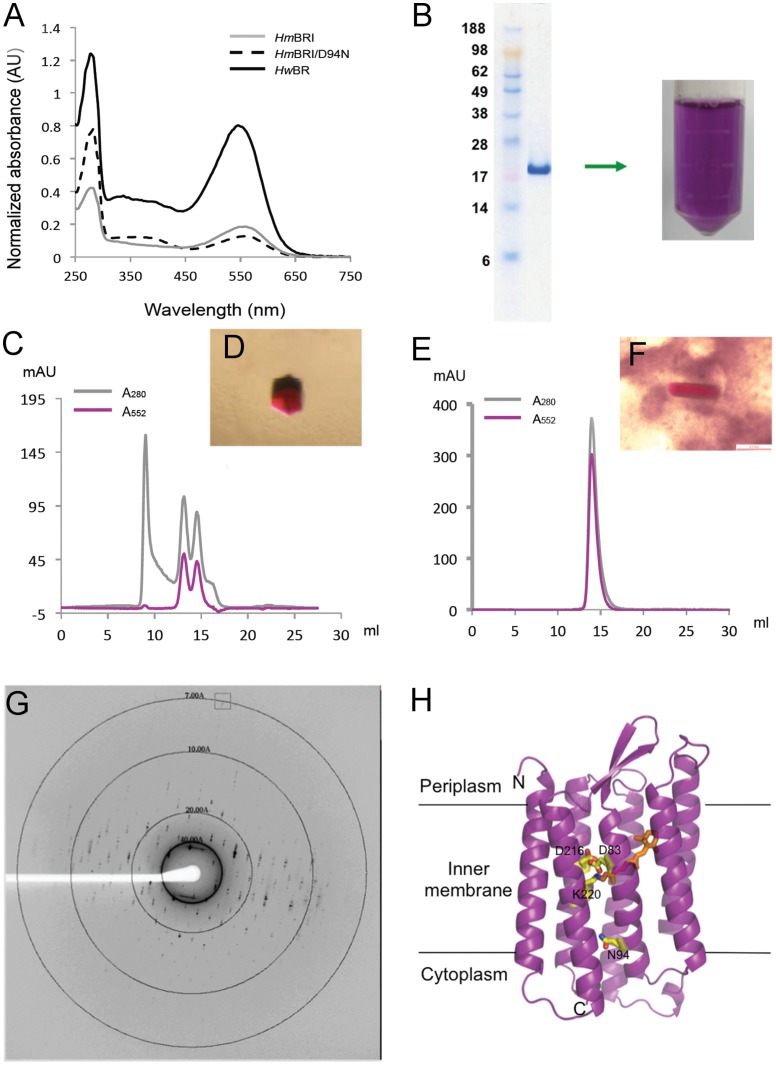
Characterization of BRs. (A) The absorbance spectrum of BRs. The UV-Vis scan was plotted from 250 to 750 nm, and the absorbance peak is at 552 nm. (B) The purified *Hm*BRI/D94N, which shows a single band on SDS-PAGE, is a purple solution. The SEC profile (C) and crystal (D) of purified *Hm*BRI/D94N and the SEC profile (E) and crystal (F) of *Hw*BR are shown. (G) The X-ray diffraction pattern and (H) The structural model of *Hm*BRI/D94N.

The UV-VIS spectra scanning of purified *Hm*BRI, D94N and *Hw*BR qualitatively showed that these BRs are functional, as revealed by their specific absorption peak at 552 nm (Figure 4A). Large-scale expression and purification of *Hm*BRI/D94N showed a single band by SDS-PAGE (Figure 4B). To obtain high-quality proteins, achieving a single elution peak monodispersed from a size exclusion column (SEC) is essential for membrane protein structural determination. The SEC result of purified *Hm*BRI/D94N showed that some aggregated opsin (rhodopsin without retinal) in the void volume, and two conformations of this protein equilibrated simultaneously (Figure 4C). The robotic screened crystallization conditions of purified *Hm*BRI/D94N in 50 mM MES, pH 6.5, 150 mM NaCl, and 0.2% DM using vapor diffusion method were found in several commercial kits for membrane proteins (Molecular Dimensions, Inc.). The hexagonal purple crystals (Figure 4D) grown in 50 mM sodium acetate, pH 4.2, 30% PEG600 and 0.2 M calcium chloride showed the best X-ray diffraction, up to 7 Å resolution, using BL44XU at SPring-8 (Figure 4G). Even though we cannot obtain the *Hm*BRI/D94N structure due to low-resolution data and detergent packing in the crystal (strong diffraction at approximately 40 Å), a homologous model using *Hs*BR (1C3W) as the template was obtained (Figure 4H). The N terminus of *Hm*BRI/D94N is located in the periplasma, while the C terminus is located in cytoplasm. The K220 residue bound with retinal through a Schiff base interaction, and the key residues charged protonation, D83, D216 and the N94 mutant are located in the center channel of the protein. Interestingly, the purified *Hw*BR presented a monodispersed peak in 50 mM sodium acetate, pH 4.5, 150 mM NaCl, and 0.2% DM after a series of buffer and detergent screens (Figure 4E). Using crystallization screening kits, the hexagonal purple crystals (Figure 4F) grown in 0.2 M calcium chloride, 0.1 M Tris-HCl, pH 8.0, 44% PEG400 showed X-ray diffraction pattern to 10 Å resolution (data collected using in-house Rigaku FR-E X-ray generator). Otherwise, *Hw*BR has the highest expression level of wild-type BRs under our system, and studies of its functions and the properties as a fusion tag are ongoing.

### Characterization of the Target Integral Membrane Proteins UppP and CaiT

**Figure 5:**
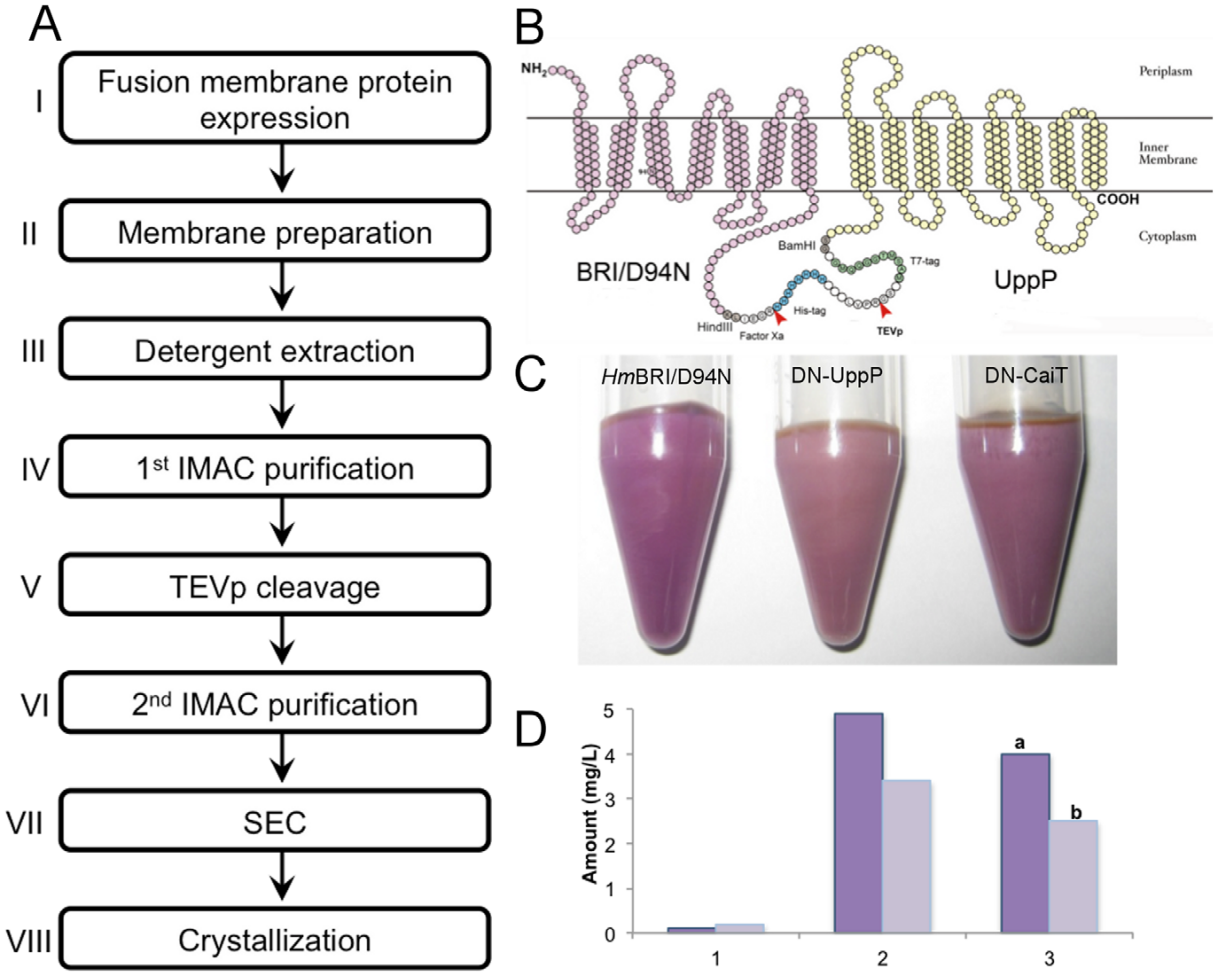
Fusion protein expression. (A) A flow chart illustrating the purification strategy for the *Hm*BRI/D94N fusion membrane protein. Step I: *E. coli* C43 or C41(DE3) expression. Step II: Collection of the purple membrane fraction by ultracentrifugation. Step III: Detergent extraction of the purple membrane. Step IV: Application of the purple protein solution to the 1^st^ IMAC column and collection of the eluted fusion protein. Step V: TEV protease digestion of the fusion protein. Step VI: Reverse IMAC column purification, with the target protein collected in the flow-through. Step VII: Size exclusion column (SEC) chromatography, resulting in a monodispersed peak. Step VIII: Crystallization. (B) Topology prediction for the *Hm*BRI/D94N-UppP fusion protein. (C) Expression cell pellets of *Hm*BRI/D94N (DN), DN-UppP and DN-CaiT. (D) Expression level of UppP (dark purple bar) and CaiT (light purple bar). 1. Gene constructed in pET51. 2. Protein purified from DN fusion system. 3. Protein expression in literature. ^a^Data from Ghachi et al., 2004. ^b^Data from Vinothkumar et al., 2006.

Most membrane proteins function as channels, receptors, transporters and signal transduction elements. To examine whether the target protein obtained in our *Hm*BRI/D94N fusion tag system adopts the correct fold, we selected the integral membrane enzymes undecaprenyl pyrophosphate phosphatase (UppP), and carnitine/butyrobetaine antiporter (CaiT), as they both could be assayed, as the target membrane proteins. Based on our cloning strategy, a flowchart for the purification of each target protein using the fusion purification system was established (Figure 5A). To enhance the binding affinity of the fusion protein, we introduced a 12×His tag. The fusion protein was eluted using 500 mM imidazole during the first IMAC column purification step. In the fusion protein, downstream from the His tag for affinity column binding, we introduced a TEVp cleavage site at the N-terminus of the target protein to obtain the native form by proteolytic digestion. After cleavage with TEVp, a second IMAC column was used to remove the uncleaved fusion protein and the His tagged TEVp and *Hm*BRI/D94N. The target protein was collected in the flow-through fractions. Since SEC was commonly used in membrane protein purification for obtaining homogeneous membrane protein. Here we used Superdex 200 HR 10/30 column for the final step of purification. The topology diagram predicted for the fusion membrane protein-*Hm*BRI/D94N-UppP, which facilitated the visualization of the purification steps, is shown in Figure 5B.

The expression pellets for the *Hm*BRI/D94N, -UppP and -CaiT fusions all appeared purple in color (Figure 5C). Compared to the expression levels when using pET51 expression vector without a fusion tag (0.1 and 0.2 mg/L), expression of UppP and CaiT using the fusion system enhanced the overall expression level by 50- and 17-fold (4.9 and 3.4 mg/L), respectively. The amounts of purified UppP and CaiT were still greater than the amounts reported in previous studies (4 and 2.5 mg/L) [31], [32] (Figure 5D).

**Figure 6:**
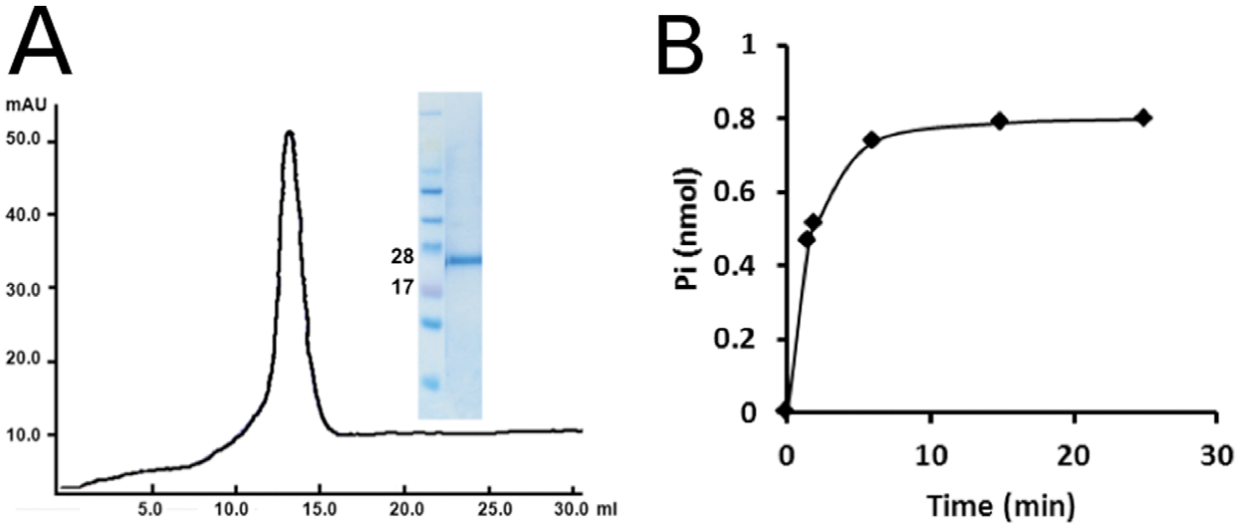
Purification and functional assay of UppP. (A) The monodispersed peak fraction at the final step of UppP purification showed a single band on the SDS-PAGE. (B) The time dependence of UppP phosphatese activity was presented using Fpp as the substrate.

The purified UppP showed a monodispersed peak by SEC and a single band by SDS-PAGE (Figure 6A). For testing the phosphatase activity of UppP, assays were performed in the presence of various substrates using Malachite Green assay. Since the intended substrate, Upp, was difficult to synthesize and purify [31], we used farnesyl pyrophosphate (Fpp) as a model substrate. Touze *et al*. [33] previously reported that another *E. coli* undecaprenyl pyrophosphate phosphatase, PgpB, which has no sequence homology with UppP, catalyzed the dephosphorylation of Upp with a relatively low efficiency compared to diacylglycerol pyrophosphate and farnesyl pyrophosphate lipid substrates. In our activity assay test, Fpp was a suitable substrate for UppP. A time-dependent phosphatase assay showed that purified UppP was a functional protein capable of releasing free phosphate (Figure 6B).

**Figure 7:**
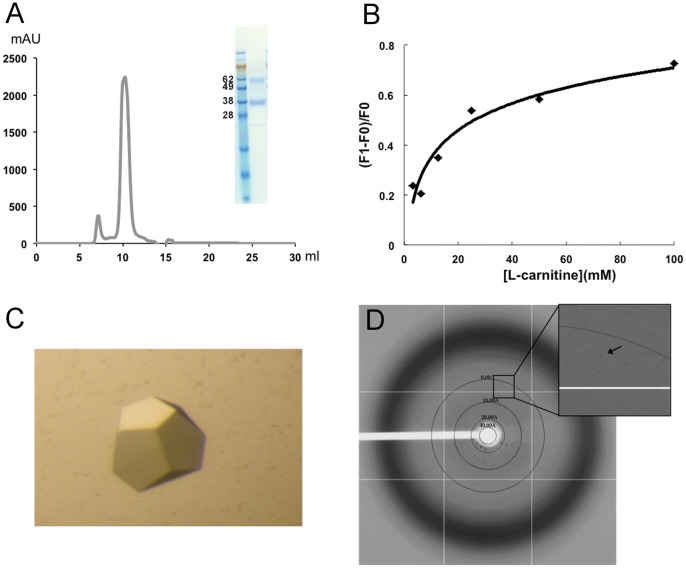
Characterization of CaiT. (A) Size exclusion column of CaiT purification and SDS-PAGE. (B) Binding of L-carnitine in CaiT. (C) Crystal of CaiT. (D) X-ray diffraction of the crystal with resolution up to 6.5 Å where the arrow shows.

The purified CaiT showed a monodispersed SEC peak with a major monomer band and a minor dimer band on the SDS-PAGE (Figure 7A). The SDS-PAGE result is consistent with those reported in literatures [32], [34]. The binding affinity of L-carnitine in CaiT was shown in Figure 7B. We grew crystals of the functional CaiT (Figure 7C), and the best spot of x-ray diffraction is up to 6.5 Å (Figure 7D) and the diffraction data are shown in Table S1. To optimize the crystallization of CaiT, we need to co-crystalize the protein with carnitine and add other detergents, e. g., OG and Cymol-5. The identity of the purified UppP and CaiT had been confirmed using in-gel digestion for mass spectrometry (Figure S1).

## Discussion

Membrane proteins are key drug targets but they are often difficult to purify, express and analyze. Establishing a high-throughput heterologous expression system is important for membrane protein research. Currently, *E. coli* is the simplest low-cost expression host, and several high-throughput *E. coli* expression systems for membrane proteins have been published in the literature [6]. In addition, a membrane protein expression system featuring a C-terminal GFP tag, which serves as an indicator of protein folding, has been developed. However, this monitoring system requires fluorescence excitation and cannot influence the expression level of membrane proteins [12], [35]. Even cell-free protein expression systems-requiring detergents, reconstitution or direct insertion into vesicles and liposomes, and the use of nanolipoprotein particles-have been applied in difficult-to-express membrane proteins [3].

A useful and specific membrane protein fusion tag should be established to facilitate future research efforts. Based on homology modeling study, *Hm*BRI has seven transmembrane helices and two β strands conformation bound to the chromophore retinal. Several rhodopsins from various halobacteria have been expressed with a his-tag in *E. coli*, producing unstable, inclusion body or low-yield heterologous expression of rhodopsins [36]–[38]. We found that *Hm*BRI/D94N has the highest expression yield (∼70 mg/L) when using the most common pET expression system in *E. coli* under modified expression conditions. The macroscopic *Hm*BRI/D94N appearance was useful for quick measurements of protein expression levels because it could be measured quantitatively by UV/VIS absorption. The concept of the purple BR-fusion membrane protein expression system is similar to the visible Cherry^TM^Express soluble protein expression system. *Hm*BRI/D94N is a GPCR, which normally has its N terminus at the periplasm and C terminus at the cytoplasmic site. In our system, the N terminus of the target membrane protein should be located in the cytoplasm to fold with correct topology. To test the novel macroscopic *Hm*BRI/D94N fusion tag system, UppP and CaiT were used as examples to demonstrate that our strategy indeed could produce functional proteins. Furthermore, *Hw*BR was expressed at high levels and purified with a monodispersed peak on an SEC column. Monodispersity is very important for membrane protein crystallization. A fusion tag that is able to produce a monodispersed peak can be used to assess suitable detergents and help crystal packing, similar to other systems, such as GFP and T4 lysozyme. Our results suggested that *Hw*BR might be the next-generation BR-fusion membrane protein expression tag.

In conclusion, we have designed an effective platform with several advantages for preparing integral membrane proteins. *Hm*BRI/D94N can target proteins of interest to the membrane and facilitate these membrane proteins to fold properly. Because correctly folded photoreceptors form purple membrane, we can directly visualize the expressed proteins and estimate the expression level of the fusion proteins. The expression level of the target membrane proteins may be increased, stimulated by the high expression level of *Hm*BRI/D94N. Separation of the fusion tag and the target protein can be efficiently achieved by protease digestion, yielding the target membrane protein. Using *Hm*BRI/D94N as a novel membrane protein expression tag will allow researchers to express and produce native membrane proteins.

## Materials and Methods

### Construction of Expression Plasmids

The plasmid vector p*Hmbop1*/D94N (the *bop1* is the gene encodes BRI) was constructed previously [24], and the *Hwbop1* was constructed as well (Figure 2A). The *E. coli uppP* gene was PCR-amplified from *E. coli* BL21(DE3) chromosome. BamHI and XhoI restriction sites were introduced at the 5′ and 3′ ends, respectively. The gene was then inserted into the pET28a vector (NOVAGEN). The vector for overexpression of the *Hm*BRI/D94N-UppP fusion protein was constructed as follows. The 6×His-thrombin cleavage site-T7 tag-*uppP* fragment from pET28a was subcloned into the 3′ end of *Hmbop1*/D94N in pET21b. Furthermore, several modifications were used to generate an efficient expression vector, p*Hmbop1*/D94N-*uppP*, including the exchange of a thrombin cleavage site for a Factor Xa site (IEGR↓), extension of the 6×His tag to a 12×His tag and insertion of TEV protease cleavage site (ENLYFQ↓G) to the 5′end of T7 tag. Based on the resultant plasmid, the genes encoding the target proteins could be inserted between BamHI and XhoI sites (Figure 2B). For construction of *caiT*, we inserted a Strep tag at the 3′ end followed by *caiT* gene, which was constructed between NdeI and XhoI sites to assist in additional purification steps.

### Protein Expression and Purification

The expression vectors harboring the *Hmbop1*/D94N, *Hwbop1*, *Hmbop1*/D94N-*uppP* and -*caiT* genes were transformed into *E. coli* C43(DE3) which were grown at 37°C in LB medium containing 100 µg/ml ampicillin. When the optical density of the culture reached an OD_600_ of 1.0, the protein expression was induced for 5 hrs by the addition of 0.5 mM isopropyl-β-D-thiogalactoside (IPTG) and 5–10 µM *all-trans* retinal (Sigma) at 37°C. For purification of *Hm*BRI/D94N and *Hw*BR, we directly treated the disrupted cell lysate in 50°C water bath for 30 mins. The pellet with the membrane fraction was obtained by centrifugation at 20,000 rpm for 30 mins. After extracting the protein from membrane fraction using buffer A (50 mM Tris, pH 7.5, 150 mM NaCl) with 2% DM overnight, we purified the protein by Ni-NTA column and eluted by buffer A containing 500 mM imidazole and 0.2% DM. The purified protein was finally dialyzed against 50 mM MES, pH 6.5, 150 mM NaCl and 0.2% DM.

For the purification of UppP and CaiT, the cells were harvested and re-suspended in buffer A. The cells were disrupted by Constant Cell Disruption Systems (Constant Systems Ltd), and the membrane and soluble proteins were separated by ultracentrifugation at 100,000×*g* for 1.5 h. The resulting pellet was solubilized by incubation in buffer A supplemented with 20 mM imidazole and 1% (w/v) DDM detergent overnight at 4°C. The latter solution was centrifuged (35,000 rpm for 1 hr at 4°C in a Beckman Ti45 rotor), and the supernatant was loaded onto Ni-NTA column and washed with buffer A containing 20 mM imidazole and 0.05% DDM. TEV protease digestion was performed after exchanging the buffer into buffer A, and the reaction mixture was incubated at 4°C overnight.

The native UppP was eluted by washing with buffer A containing 0.05% DDM. Size exclusion chromatography (SEC) was performed using a Superdex 200 HR 10/30 column (GE Healthcare) equilibrated with two column volumes of buffer A with 0.05% DDM. Elution of the proteins was followed at 280 nm. A 500 µl aliquot of the protein sample was loaded onto the column at a flow rate of 0.3 ml/min.

For CaiT purification, a Strep-tag™ (GE Healthcare) purification step was further introduced and performed prior to the SEC step.

### Detergent Screening

Commonly used detergents-DM, UDM, DDM, OG, NG and LDAO- were prepared in buffer A at concentrations of 1∼2%. The expressed *Hm*BRI/D94N *E. coli* cells were disrupted by Constant Cell Disruption Systems (Constant Systems Ltd). One milliliter aliquots of the suspension were transferred into 1.5 ml tubes and incubated at 50°C for 30 mins. The membranes containing the purple *Hm*BRI/D94N were harvested and centrifuged for 10 mins at 13,000 rpm. The detergent solutions (1 ml each) were added into the tubes and incubated at 4°C for at least 1 hr to solubilize the membranes. The solutions were centrifuged at 13,000 rpm for 10 mins to discard the insoluble components. The supernatants were analyzed by UV-VIS spectroscopy at 280 nm and 552 nm using Nanodrop 1000 spectrophotometer (Thermo). All detergent tests were performed in triplicate.

### Protein Monitoring

Electrophoresis was carried out in SDS-PAGE using a NuPAGE electrophoresis system (Invitrogen) and conditions were prepared for membrane proteins. Protein concentration was quantified by UV-VIS spectroscopy at 280 nm and 552 nm using Nanodrop 1000 spectrophotometer (Thermo).

### Phosphatase Assay

UppP activity was determined by the Malachite Green assay using Phosphate Colorimetric Assay Kit purchased from BioVision. The enzymatic assay reaction mixture (200 µl) containing 50 mM Tris-HCl, pH 7.5, 150 mM NaCl, 0.2% DDM, 100 µM substrate (Fpp) and appropriately diluted purified UppP was incubated at 37°C. The reaction was quenched by adding 30 µl of Malachite Green Reagent and then the tubes were incubated for 30 min at room temperature. After incubation, the reaction mixture was transferred to 96-well plates and the released phosphate that reacted with Malachite Green was measured at 650 nm according to the reaction time course. The amounts of released phosphate were quantified relative to a phosphate standard curve.

### CaiT Activity Assay

The CaiT activity assay was modified from the method described previously [39]. The fluorescence intensity measured at 339.50 nm was used to calculate the apparent affinity constant, *K*_0.5_, which reflects the ligand concentration, causing a half-maximum change in CaiT fluorescence.

### Crystallization and X-ray Diffraction Data Collection

The *Hm*BRI/D94N crystals were grown at 20°C using sitting drop vapor diffusion method. The protein (∼3 mg/ml) was mixed 1∶1 with a reservoir solution composed of 50 mM HEPES, pH 7.8 and 26% (v/v) PEG600. The X-ray diffraction datasets were collected at BL44XU, SPring-8, Japan and processed using HKL2000 [40]. The *Hw*BR crystals were grown at 20°C using sitting drop vapor diffusion method. The protein (∼10 mg/ml) was mixed 1∶1 with a reservoir solution composed of 0.1 M Tris-HCl, pH 8.0, 0.2 M calcium chloride and 44% (v/v) PEG400.

We grew native CaiT crystals using the sitting-drop vapor-diffusion method at 20°C by mixing an equal volume of 5.7 mg/ml of the protein in buffer A with 0.05% DDM with a reservoir solution composed of 50 mM magnesium acetate, pH 4.5, 26%–28% (v/v) PEG400, and 50–200 mM NaCl. We collected X-ray diffraction datasets on a ADSC Quantum315 CCD-detector at the BL13B beamline at the National Synchrotron Radiation Research Center (NSRRC), HsinChu, Taiwan. We obtained the phases by molecular replacement using *E. coli* CaiT complexed with γ-butyrobetaine as template (2WSX). PHENIX and COOT programs are used for molecular replacement and structure viewer.

## Supporting Information

Figure S1 **Protein identification of purified UppP and CaiT using MASS spectrometry.**
(DOCX)

Table S1 **Data collection statistics of** ***Hm*** **BRI/D94N and CaiT.**
(DOCX)
