# Supplementary material for: Using Haloarcula marismortui Bacteriorhodopsin as a Fusion Tag for Enhancing and Visible Expression of Integral Membrane Proteins in Escherichia coli
Source: PLoS One. 2013 Feb 15;8(2):e56363. doi: 10.1371/journal.pone.0056363 (PMC3574148; doi:10.1371/journal.pone.0056363)
Supplement: Figure S1 — Protein identification of purified UppP and CaiT using MASS spectrometry. (DOCX) [file pone.0056363.s001.docx]

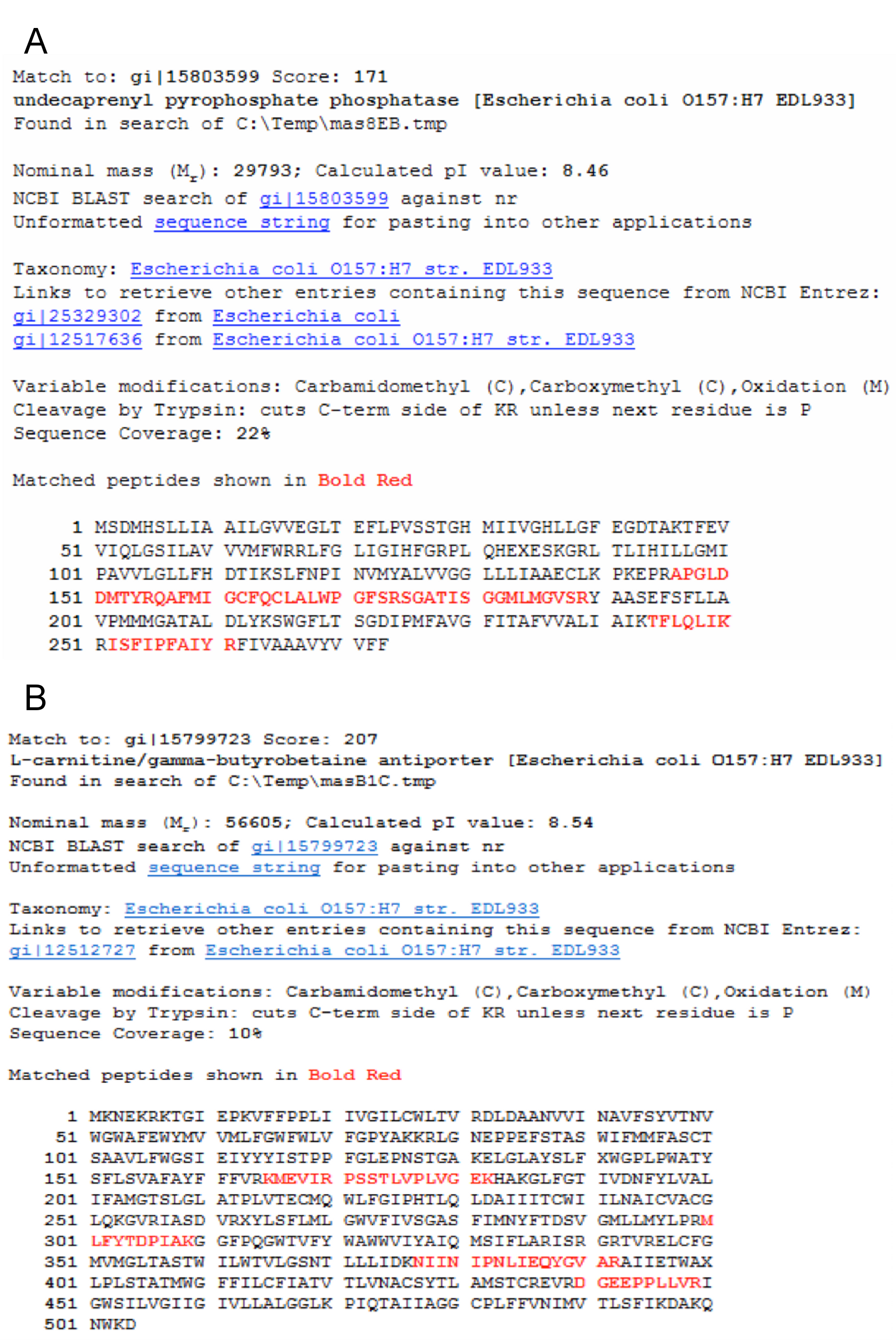


**Figure S1. MALDI-TOF mass spectrum obtained from single bands of purified UppP and CaiT after trypsin digestion.** (A) UppP and (B) CaiT peptide mass fingerprint data, and the matched peptides are shown in red.
