# Supplementary material for: Using Haloarcula marismortui Bacteriorhodopsin as a Fusion Tag for Enhancing and Visible Expression of Integral Membrane Proteins in Escherichia coli
Source: PLoS One. 2013 Feb 15;8(2):e56363. doi: 10.1371/journal.pone.0056363 (PMC3574148; doi:10.1371/journal.pone.0056363)
Supplement: Table S1 — Data collection statistics of Hm BRI/D94N and CaiT. (DOCX) [file pone.0056363.s002.docx]

| **Table S1 Data collection statistics** | | |
| --- | --- | --- |
| Sample name | HmBRI/D94N | CaiT |
| Beamline | 44XU, SPring-8 | 13B1, NSRRC |
| Wavelength (Å) | 1.5 | 1 |
| Space group | *P*622 | *P*3_2_ |
| Cell dimensions  *a, b, c* (Å)  γ (°) | 185, 185, 585  120 | 131, 131, 156  120 |
| Resolution (Å) | 34.0-8.7 (8.85-8.70) | 30.0-7.0 (7.25-7.00) |
| R_merge_ | 0.10 (0.58) | 0.045 (0.64) |
| I/σI | 34.0 (7.3) | 41.8 (2.8) |
| Completeness (%) | 96.0 (97.7) | 99.9 (100.0) |
| Redundancy | 11.3 (12.0) | 7.1 (6.9) |
